# Supplementary material for: Malnutrition Aggravates Alterations Observed in the Gut Structure and Immune Response of Mice Infected with Leishmania infantum
Source: Microorganisms. 2021 Jun 11;9(6):1270. doi: 10.3390/microorganisms9061270 (PMC8230684; doi:10.3390/microorganisms9061270)
Supplement: Supplementary file 1 [file microorganisms-09-01270-s001.zip › Legends to supplementary figures.pdf]

Legends to supplementary figures

Supplementary figure 1. Analysis of IgA distribution in the duodenum of malnourished BALB / c mice infected with *Leishmania infantum*. (A) Representative images of tissue IgA (green) detected by immunofluorescence in sections of duodenum from different experimental groups. The cell nuclei were marked with DAPI (blue). $n = 4$ animals per group. 10X increase. (B) Quantitative analysis of tissue IgA+ area. IgA levels are expressed as a percentage of the marked area. Significant differences due to infection (b) $p < 0.001$, and interaction of diet and infection (c) $p < 0.01$.

Supplementary Table S1. Sequences of primers used for real time qPCR

Supplementary Table S2. Score values for histological alterations in the intestinal tissue
