# Supplementary material for: Malnutrition Aggravates Alterations Observed in the Gut Structure and Immune Response of Mice Infected with Leishmania infantum
Source: Microorganisms. 2021 Jun 11;9(6):1270. doi: 10.3390/microorganisms9061270 (PMC8230684; doi:10.3390/microorganisms9061270)
Supplement: Supplementary file 1 [file microorganisms-09-01270-s001.zip › Supplementary Table 1.pdf]

Supplementary Table S1. Sequences of primers used for real time qPCR

Accession Number	Target	Forward Primer	Reverse Primer	Product size (bp)	Slope	Efficiency (E) (%)	Correlation coefficient (R^2)
NM_010548.2	***IL-10***	ACAACATACTGCTAACCGACTCCT	GGCAACCCAAGTAACCCTTAAAGT	57	3.15	108	0.97
NM_008351.3	***IL-12***	CTCTCATATTCACTATACAAGTTG	GCTCTTCTGCTAACACAT	94	3.29	101	0.80
NM_010552.3	***IL-17A***	TCTGTTCTCATCCAGCAAGA	ATCTTCTCGACCCTGAAAGT	80	3.27	102	0.94
NM_008337.3	***IFN-γ***	AGGACACAACAAGATGGA	TTAGTGAGAGTGAACATTACAG	141	3.32	100	0.92
NM_001278601.1	***TNF-α***	CTTCCTACCTTCAGACCTT	GCCTTCCAAATAAATACATTCAT	153	2.60	120	0.97
NM_011577.1	***TGF-β***	ATTCCTGGCGTTACCTTGG	CCTGTATTCCGTCTCCTTGG	117	2.46	155	0.75
NM_025567.2	***Cyc1***	GGTGTCATTGCGAGAAGG	GGTGCCATCATCATACTCC	106	3.32	100	0.99
NM_016774.3	***ATPβ5***	TGAGTGTTGAGCAGGAGATTC	TTGGCGACATTGTTGATTAGC	148	3.39	97	0.99
NM_013556.2	***HRTP***	CGTGATTAGCGATGATGAACC	AAGTCTTTCAGTCCTGTCCA	124	3.00	107	0.99
AF285161.1	***UBC***	CTGTGAGAGCCGTGGATATTGG	GCACTTCCGTCTTTCAGCAAA	84	3.47	94	0.98
AF169140.1	***kDNA***	TGTTGGTTGTGTGACTTTA	CATCCCACCAGACTAATC	74	2.99	115	0.99
