# Supplementary figures and images for: Malnutrition Aggravates Alterations Observed in the Gut Structure and Immune Response of Mice Infected with Leishmania infantum
Source: Microorganisms. 2021 Jun 11;9(6):1270. doi: 10.3390/microorganisms9061270 (PMC8230684; doi:10.3390/microorganisms9061270)

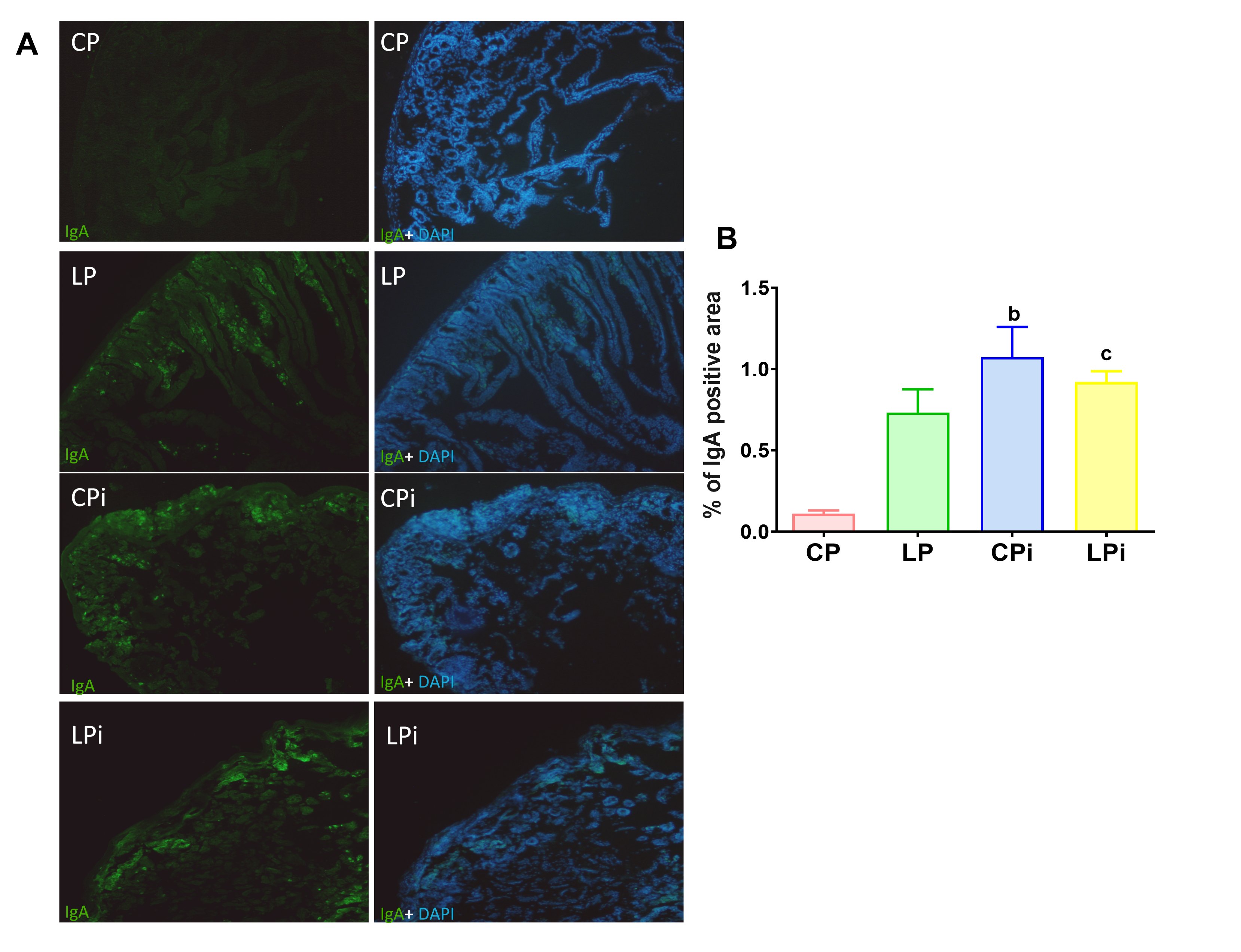

Supplement: Supplementary file 1 [file microorganisms-09-01270-s001.zip › Supplementary figure 1.tif]
